# Prevalence of xerostomia and its association with systemic diseases and medications in the elderly: a cross-sectional study

**DOI:** 10.1590/1516-3180.2020.0616.R3.1902021

**Published:** 2021-06-25

**Authors:** Cindel Balbinot Fornari, Daniel Bergonci, Cauane Bruna Stein, Bernardo Antonio Agostini, Lilian Rigo

**Affiliations:** I Undergraduate Student, Dentistry School, Faculdade Meridional (IMED), Passo Fundo (RS), Brazil.; II Master’s Student, Dentistry School, Faculdade Meridional (IMED), Passo Fundo (RS), Brazil.; III Undergraduate Student, Dentistry School, Faculdade Meridional (IMED), Passo Fundo (RS), Brazil.; IV PhD. Professor, Dentistry School, Faculdade Meridional (IMED), Passo Fundo (RS), Brazil.; V PhD. Professor, Dentistry School, Faculdade Meridional (IMED), Passo Fundo (RS), Brazil.

**Keywords:** Chronic disease, Diabetes mellitus, Drug effects [subheading], Aged, Xerostomia, Elderly, Diabetes, Effect of drugs, Hyposalivation, Dry mouth

## Abstract

**BACKGROUND:**

Dry mouth syndrome or xerostomia is defined as decreased salivary flow or hypofunction of salivary glands. Its origins are multicausal and might be the result of a change in the salivary glands or a systemic imbalance.

**OBJECTIVE:**

To ascertain the prevalence of self-reported xerostomia and to identify associated factors.

**DESIGN AND SETTING:**

Cross-sectional study on the entire population of 293 elderly people over 60 years of age living in a Brazilian municipality.

**METHODS:**

Data were gathered from a questionnaire that asked about demographic data, chronic diseases and use of continuous medications, and which used the Xerostomia Inventory (XI) to evaluate dry mouth sensation. Our analysis consisted of multivariate regression and estimation of odds ratios (OR) and their respective 95% confidence intervals (CI) in binary logistic regression models.

**RESULTS:**

The prevalence of self-reported xerostomia was 19.1%. Elderly people with diabetes had higher odds of having self-reported xerostomia (OR: 3.59; 95% CI: 1.48-8.68; P < 0.001) as did those who had chronic diseases and used continuous medication (OR: 2.3; 95% CI: 1.19-4.67; P = 0.009). Elderly people who used continuous medication for the gastrointestinal tract were more likely to have xerostomia (OR: 2.14; 95% CI: 1.03-1.44; P = 0.030).

**CONCLUSIONS:**

Elderly people with diabetes and chronic diseases who were using continuous medication were more likely to have dry mouth. Use of continuous medications for the gastrointestinal tract led to a greater chance of having self-reported xerostomia.

## INTRODUCTION

Saliva plays an important role in oral health. Besides being involved in protection against bacteria and fungi, it transports nutrients and digestive enzymes, lubricates the mucosa, facilitates mastication, swallowing and speech, and acts in the process of tooth remineralization.^1^^,^^2^ Saliva is produced by salivary glands, which are exocrine, such as the parotid, submandibular and sublingual glands. These are the most important pairs of glands and are responsible for 95% of saliva production.^3^ In addition, there are other smaller-sized glands throughout the oral cavity, lips and tongue, which help in the process of salivation. These structures produce saliva at certain moments and respond to a series of sensory, taste and olfactory stimuli.

The volume of saliva production may vary according to stimulation. The salivary flow is greater after meals and lower during sleep. In healthy individuals, there is, on average, 1000 ml to 1500 ml of saliva secretion in a day.^3^ However, there are several consequences of decreased salivary flow. These include some diseases and problems such as cavities, periodontal disease, various infections, dysphagia, halitosis and difficulties with the stability of dental prostheses.^4^

Dry mouth syndrome or xerostomia is defined as decreased salivary flow or hypofunction of the salivary glands.^5^ Its origins are multicausal and might be the result of a change in the salivary glands or a systemic imbalance.^5^^,^^6^^,^^7^ Some determinants such as continuous use of medication, radiation, systemic diseases and factors common to aging might be associated with a dry mouth condition.^8^ Xerostomia is not considered to be a disease but, rather, a manifestation of a series of pathological conditions that considerably alter patients’ quality of life. It can affect chewing, swallowing, use of prostheses and speech.^9^ Villa et al. reported that xerostomia that is secondary to hyposalivation may also result in fungal infections such as candidiasis, tooth decay, halitosis, changes to the sense of taste and a burning sensation in the mouth.^10^ In addition, they reported that xerostomia may be a consequence of head and neck radiotherapy, depression, anxiety, stress and even malnutrition. It needs to be emphasized that some of the most common chronic conditions today are symptoms of depression and anxiety.^10^

There is some evidence that certain chronic diseases might be determinants of xerostomia and/or hyposalivation.^11^^,^^12^^,^^13^^,^^14^^,^^15^ One of the diseases most investigated today is diabetes mellitus (DM), a chronic disease characterized by hyperglycemia and insufficient insulin production by the pancreas.^8^ The insulin that is produced has the function of regulating carbohydrate metabolism, and its absence causes glucose to remain in the bloodstream, thus characterizing a state of hyperglycemia. Hyposalivation in patients with uncontrolled diabetes might be caused by an increase in diuresis, which could affect saliva production,^16^ but it might also be caused by a condition called neuropathy.^17^^,^^18^^,^^19^^,^^20^ Therefore, there still seems to be much doubt concerning the cause-and-effect relationship between this pathological condition and xerostomia.^21^

In addition to correlations with chronic diseases, a relationship between xerostomia and use of continuous medication has been highlighted. Wiener et al.^22^ and Van der Putten et al.^23^ added anticholinergic, diuretic and antipsychotic drugs to the list of associated factors. Freitas et al.^6^ also included some types of analgesic and xerostomia-associated antibiotics.

Xerostomia is an important condition but is still little-known by the population and has been neglected by health professionals. In addition, studies on this condition are scarce and unenlightening. The absence of explanations about the determining factors of this condition can be highlighted, especially in relation to the non-institutionalized elderly population. The prevalence of xerostomia in the population has been reported to range from 5.5% to 46%.^10^ Gender and age-related differences have been observed, such that older individuals may have more symptoms of xerostomia. However, this could be due to the higher number of xerogenic drugs used to treat chronic diseases and might not only be related to age.^10^^,^^21^ Nonetheless, these associations still seem unclear.

## OBJECTIVE

Based on these scientific data, the objective of the present study was to ascertain the prevalence of self-reported xerostomia and to identify its association with chronic diseases, continuous medication use, age and gender, among elderly people in a municipality in southern Brazil.

The hypothesis for this study was that the presence of chronic systemic diseases and use of medications among the elderly people investigated would show associations with self-reported xerostomia.

## METHODS

This study was submitted to and approved by the Research Ethics Committee of Faculdade Meridional (IMED), under the approval no. 2,711,544 and CAEE 90966718.0.0000.5319, on June 13, 2018, in accordance with the rules of resolution 466/12. All individuals participating in the research signed an informed consent statement in which they agreed to be part of it. Care was taken in this study to ensure confidentiality regarding identity and privacy, and also the confidentiality of the data obtained.

The present work was compiled in accordance with the recommendations of the STROBE statement (Strengthening the reporting of observational studies in epidemiology).^24^

### Design, sample and location of study

The present study took a quantitative approach, with a cross-sectional design. The study population consisted of the elderly population of the municipality of Vanini, which is located in the northwest of the state of Rio Grande do Sul. It has an estimated total population of 2,104 inhabitants and a total area of 69.9 km^2^.^25^

The population of Vanini over 60 years of age consisted of 300 people.^25^ All of this population was eligible, but seven people were excluded from the study (see criteria, below). Thus, the study population was formed by 293 elderly people aged 60 years and over. There were no losses in this study.

### Inclusion and exclusion criteria

All the elderly population aged 60 years and over participated in this study. Only those with neurological conditions and patients with head and neck cancers undergoing radiotherapy were excluded from the study.

### Data collection procedures

Data collection was performed through home visits to all houses in the municipality, made by a team composed of four students of the dentistry course, between August and September 2018. First, a pilot test was performed on twenty elderly people in order to train the researchers to collect data and to check for possible doubts or problems relating to completion of the research instrument by the elderly subjects, thus minimizing possible bias in the research methodology. However, there were no changes to the procedures and, later on, these data from the pilot study were included in the final sample.

The data relating to the variable of self-reported xerostomia were collected by means of a validated questionnaire for xerostomia and dry mouth sensation that is used to verify cases of self-reported xerostomia. The Xerostomia Inventory (XI) includes eleven items.^26^ A previous study validated this questionnaire for use among Brazilian individuals.^27^ Each item in this questionnaire has five response options: never, hardly ever, occasionally, fairly often and very often. The questions are the following: “Do you have difficulties swallowing any foods?”, “Do you have difficulties eating dry foods?”, “Does your mouth feel dry when eating a meal?”, “Does your nose feel dry?”, “Does your face feel dry?”, “Do you suck sweets or cough lollies to relieve dry mouth?”, “Do you get up at night to drink?”, “Do your eyes feel dry?”, “Do your lips feel dry?”, “Does your mouth feel dry?” and “Do you sip liquids to aid in swallowing food?”,

We added questions asking for demographic data (gender and age) and questions asking about chronic diseases and continuous medication use, consisting of the following: “Do you use continuous medications?”, “Do you have diabetes, thyroid dysfunction, rheumatoid arthritis, depression and/or anxiety, HIV, hypertension and/or any other diseases?” and “Do you use medications for the stomach, cholesterol or diuretics, or do you use anticoagulants?” In addition, we also included a question on “Difficulty in using a dental prosthesis”.

### Outcome variable

We composed the outcome variable of this study based on studies conducted by Thomson et al. in 1999^26^ and 2006,^28^ which used a single question from the XI questionnaire to ascertain the prevalence of xerostomia. In the present study, we did not perform an oral clinical evaluation.

We combined the responses to the questions of the XI questionnaire into negative answers (no) = never, hardly never and occasionally; and positive answers (yes) = fairly often and very often. Thus, people who answered positively to the XI question “My mouth feels dry” were included in the self-reported xerostomia group.^28^

### Covariables

The variables considered were gender (female or male), age (60 to 69 years old or 70 years old or over), diabetes (yes/no), thyroid dysfunction (yes/no), rheumatoid arthritis (yes/no), depression and/or anxiety (yes/no), arterial hypertension (yes/no), HIV (yes/no), other chronic diseases and continuous medication (yes/no), use of medicine for cholesterol (yes/no), use of gastrointestinal tract medication (yes/no), use of diuretic (yes/no), use of anticoagulants (yes/no) and difficulty in using a dental prosthesis (yes/no).

The variable “other chronic diseases and continuous medication” (yes/no) was composed of diseases other than the most prevalent diseases mentioned previously and was considered together with use of drugs to control these diseases. This formed a reliable way of knowing whether the elderly individual was being medicated. The diseases included in this variable were fibromyalgia, Parkinson’s disease, hypothyroidism, osteoporosis, cardiac arrhythmia and multiple sclerosis.

### Data analysis

For the data analysis, we performed descriptive analyses and bivariate and multiple regressions. In the multiple analysis, we estimated odds ratios (OR) and their respective 95% confidence intervals, with crude variables and variables adjusted for exposure in binary logistic regression models (P-value < 0.05). The data were analyzed using the Statistical Package for the Social Sciences (SPSS) software, version 20.0 (IBM, Armonk, New York, United States).

## RESULTS

Table 1 describes the results regarding demographic variables, chronic diseases, difficulties and use of continuous medications. Most of the elderly people were between 60 and 69 years old, and a majority of the population (56%) was male.

**Table 1.** Description of demographic variables, chronic diseases and use of continuous medications among elderly people in the municipality of Vanini, Brazil, 2019 (n = 293)

Variables	n	%
**Age**		
60-69 years	175	59.7
70 or over	118	40.3
**Gender**		
Female	127	43.3
Male	166	56.7
**Diabetes**		
Yes	37	12.6
No	256	87.4
**Thyroid dysfunction**		
Yes	30	10.2
No	263	89.8
**Rheumatoid arthritis**		
Yes	8	2.7
No	285	97.3
**Depression and/or anxiety**		
Yes	68	23.2
No	225	76.8
**HIV**		
Yes	6	2.0
No	287	98.0
**Hypertension**		
Yes	167	57.0
No	126	43.0
*Other chronic diseases and continuous medication		
Yes	118	40.3
No	175	59.7
**Use of medicine for cholesterol**		
Yes	80	27.3
No	213	72.7
**Use of gastrointestinal tract medication (antacids/H2/ IPB antagonists)**		
Yes	50	17.1
No	243	82.9
**Use of diuretic**		
Yes	92	31.4
No	201	68.6
**Use of anticoagulants**		
Yes	50	17.1
No	243	82.9
**Difficulty in using dental prosthesis**		
Yes	6	2.0
No	287	98.0

*Other chronic diseases/medication – fibromyalgia, Parkinson’s disease, hypothyroidism, osteoporosis, cardiac arrhythmia, multiple sclerosis.

Among these elderly people, 12.6% presented diabetes; 10.2%, diseases relating to thyroid dysfunction; 23.2%, depression and anxiety; 57%, arterial hypertension; and 10.2%, rheumatoid arthritis. Moreover, 40.3% of them reported having other diseases, i.e. in addition to those being researched in this study. Thus, in addition to the medications used for the chronic diseases that they reported (diabetes, thyroid dysfunction, rheumatoid arthritis, depression and anxiety, HIV and hypertension), they also used cholesterol-lowering drugs (27%) and medicines for stomach and circulatory problems (17.1%). These elderly people also had other chronic diseases and used continuous medications for their control (40.3%).

Table 2 shows the results relating to Xerostomia Inventory (XI) variables. Presence of dry mouth sensation was reported by 19.1% of the participants when asked the question “Does your mouth feel dry?” Furthermore, 13.3% had difficulty in swallowing food, 14.7% sipped liquids to aid in swallowing food and 30.4% habitually got up at night to drink. In the question about having difficulty in using a dental prosthesis, only 2% of the elderly people answered yes; however, 100% of them were using some type of dental prosthesis.

**Table 2.** Distribution of responses to questions regarding dry mouth (xerostomia) from the Xerostomia Inventory (XI), among elderly people in the municipality of Vanini, Brazil, 2019 (n = 293)

Variables	n	%
**I have difficulties swallowing certain foods**		
Yes	39	13.3
No	254	86.7
**My mouth feels dry when eating a meal**		
Yes	38	13
No	255	87
**I sip liquids to aid in swallowing food**		
Yes	43	14.7
No	250	85.3
**I get up at night to drink**		
Yes	89	30.4
No	204	69.6
**I suck sweets or cough lollies to relieve dry mouth**		
Yes	40	13.7
No	253	86.3
**My eyes feel dry**		
Yes	42	14.3
No	251	85.7
**My lips feel dry**		
Yes	42	14.3
No	251	85.7
**I have difficulty in eating dry foods**		
Yes	39	13.3
No	254	86.7
**My mouth feels dry**		
Yes	56	19.1
No	237	80.9
**The skin of my face feels dry**		
Yes	26	8.9
No	267	91.1
**The inside of my nose feels dry**		
Yes	26	8.9
No	267	91.1

To perform the binary logistic regression, all variables that had an association with a P-value < 0.20 were entered into the crude model: gender, depression or anxiety, diabetes, other chronic diseases with continuous medication, gastrointestinal tract medication, cholesterol drugs and use of anticoagulants. After multivariate adjustment, the variables of diabetes, other chronic diseases and gastrointestinal tract medication remained significant (P < 0.05), but the other variables lost their associations in the final adjusted multivariate regression analysis model (Table 3). Elderly people with diabetes had higher odds of having self-reported xerostomia (OR: 3.59; 95% CI: 1.48-8.68; P < 0.001), as did those who had chronic diseases and used continuous medication (OR: 2.3; 95% CI: 1.19-4.67; P = 0.009). The elderly people who continuously used medication for the gastrointestinal tract also had higher odds of having xerostomia (OR: 2.14; 95% CI: 1.03-1.44; P = 0.030).

**Table 3.** Bivariate (crude) and multivariate (adjusted) binary logistic regression model for the self-reported xerostomia variable, among elderly people in the municipality of Vanini, Brazil, 2019

	Crude OR (95% CI)	P-value*	Adjusted OR (95% CI)	P-value**
**Age**				
60 to 69	1		-	-
≥ 70	0.95 (0.52-1.72)	0.867		
**Gender**				
Male	1			
Female	1.63(0.88-2.99)	*0.116*	1.63 (0.83-3.20)	0.152
**Difficulty in using dental prosthesis**				
No	1		-	-
Yes	2.15 (0.38-12.0)	0.380		
**Depression or anxiety**				
No	1			
Yes	0.62 (0.32-1.20)	*0.160*	0.82 (0.39-1.72)	0.614
**Diabetes**				
No	1		1	
Yes	3.12 (1.41-6.90)	*0.005*	3.59 (1.48-8.68)	< *0.001*
**Arterial hypertension**				
No	1			
Yes	0.68 (0.37-1.25)	*0.222*		-
**Thyroid dysfunction**				
No	1			
Yes	1.62 (0.68-3.87)	*0.272*	-	-
**Other chronic diseases and continuous medication**				
No	1			
Yes	2.34 (1.29-4.23)	*0.005*	2.3 (1.19-4.67)	*0.009*
**Gastrointestinal tract medication**				
No	1		1	
Yes	2.38 (1.20-4.73)	*0.013*	2.14 (1.03-1.44)	*0.030*
**Medicine for cholesterol**				
No	1			
Yes	1.99 (1.08-3.68)	0.270	-	-
**Use of diuretic**				
No	1			
Yes	1.27 (0.69-2.35)	0.440	-	-
**Use of anticoagulants**				
No	1		1	
Yes	1.86 (0.92-3.75)	*0.083*	1.17 (0.54-2.54	0.676

*Chi-square test; **Wald test (P < 0.05 - statistically significant and shown in italics). OR = odds ratio; 95% CI = 95% confidence interval; % = frequency-percentage. Adjusted according to the following variables: gender, depression or anxiety, diabetes, other chronic diseases, gastrointestinal tract medication, cholesterol medication, anticoagulant use (P < 0.05).

## DISCUSSION

The purpose of this study was to ascertain the prevalence of self-reported xerostomia and to identify its associations with chronic diseases, continuous medication use, age and gender, among the elderly, in order to learn about these data, which had never been investigated in this location. The results showed that the prevalence of self-reported xerostomia was 19.1%, through using the XI question “Does your mouth feel dry?”. It is also important to point out that 13% reported feeling “difficulties in swallowing certain foods” and 14.7% “sipped liquids to aid in swallowing food”, which are important responses for indicating the presence of xerostomia. Xerostomia is a subjective sensation of dry mouth and is assessed by asking individuals directly about their experience with this disease. According to other studies, the question “ Does your mouth feel dry” reveals the prevalence of dry mouth.^26^^,^^28^ Thus, salivary flow was not measured in this study. Dry mouth is an important condition that negatively impacts people’s daily lives, so the results presented here should be considered with caution.

The study conducted by Thomson et al. among adults and the elderly showed that the prevalence of xerostomia was 10%, with no difference between the genders.^28^ Perotto et al.^12^ evaluated 117 dental patients, among whom 24.8% reported having xerostomia, which was associated with medication use. This differed from the results found by Freitas et al.,^6^ in which 59% of the elderly subjects reported having a feeling of dry mouth during most of the day, and this sensation was associated with the medication that they were using. The estimated global prevalence of dry mouth was found to be 22% among adult and elderly individuals in a systematic review study.^29^ However, the prevalence was higher in studies conducted exclusively among elderly people, and presence of xerostomia in these studies was correlated with older age and the need for continuous-use drugs with high xerostomic potential, among which most were used to treat chronic diseases.^29^

In a study conducted by Islas-Granillo et al.,^30^ 68.3% of the elderly subjects had xerostomia. Niklander et al.,^31^ in a survey on 566 individuals, observed that 42.4% of them took some type of medication and, of these, 17.92% reported having dry mouth. According to Lopes et al.,^32^ out of 20 women surveyed, 50% reported having xerostomia. Another study, conducted by Wiener et al.,^22^ showed that among 252 elderly individuals surveyed, 28% had xerostomia associated with sociodemographic conditions, medication use and systemic conditions. The complaint of dry mouth needs to be taken seriously by healthcare professionals and, thus, individuals with this complaint should be asked about what they feel, their medical history and the medicines that they are taking, considering that indefinite-cause xerostomia is an undiagnosed systemic imbalance.

In the present study, there was a statistically significant association between self-reported xerostomia and presence of the chronic disease diabetes mellitus. The chances that an individual with DM who uses continuous medication for this condition will have xerostomia or dry mouth are 3.59 times higher than among other elderly people. It should be noted that all the elderly people in the present study were using medication at the time of data collection.

In another study, presence of xerostomia among patients with decompensated DM was explained by increased diuresis or polyuria, which could affect saliva production.^13^ Carda et al.^33^ surveyed 33 patients with type 2 DM and found that 76.4% of them had symptoms of xerostomia. However, it has been reported that it remains undetermined whether the presence of xerostomia is higher in patients with or without diabetes.^14^ In a further study, the prevalence of xerostomia among 120 elderly people diagnosed with type 2 diabetes (60 insulin-dependent individuals and 60 who did not require it) who had been undergoing treatment for at least one year using continuous medication was surveyed. Dry mouth or xerostomia was evaluated on a visual analogue scale. Among these patients, 92.5% presented hyposalivation and 49.2% had moderate to severe dry mouth or xerostomia.^15^

Although the present study did not show any significant relationships with depression and anxiety, high blood pressure or thyroid dysfunction, several other studies have highlighted this association. Thomson et al.^28^ found relationships between xerostomia and the use of antidepressants, iron supplements and analgesics. The complaint of dry mouth was more frequent among adults who were using antidepressants, and also among those using other medications such as iron and narcotic painkillers. The individuals who were taking antidepressants were 22 times more likely to feel dry mouth or proper xerostomia.^28^ Perotto et al.^12^ observed other predisposing factors for xerostomia, consisting of age 50 years and over and presence of diabetes and hypertension.

A study by Bulthuis et al.^34^ estimating the possible role of stress in salivary secretion showed a correlation between stress and xerostomia, and it was concluded that stress was related to dry mouth sensation and consequently had an impact on quality of life. A study among adults showed a strong association between xerostomia and quality of life.^35^ According to Abrão et al.,^36^ alterations such as xerostomia and hyposalivation are common in rheumatic diseases, such that xerostomia affects 1% of patients with rheumatoid arthritis. That study evaluated 604 patients with rheumatological disorders and showed that 43% of them had hyposalivation; this hyposalivation and dry mouth/xerostomia increased with increasing severity of the rheumatological condition.^36^

In the present study, there was an association with the presence of other chronic diseases in the survey questionnaire and their continuous medication. These elderly individuals were 2.3 more likely to have self-reported xerostomia, with a prevalence of 42%. Thus, it can be inferred that, in addition to chronic diseases, the continuous medication itself might have been the cause of these results. Korn et al.^5^ observed that various systemic disorders could cause xerostomia or the feeling of dry mouth, among them Sjögren’s syndrome. Regarding the results from the present study, it is important to stress that the amount of medication used by the elderly individuals was not investigated, given that there could have been significant differences relating, not to other diseases, but to the higher number of drugs used to treat different chronic illnesses.^5^

Chronic diseases are the ones that most affect the elderly, and they lead to use of large-scale continuous medication.^37^ In addition to medications for diabetes, depression and anxiety, others can be considered, such as medications for cardiovascular diseases, nervous system disorders, gastrointestinal tract diseases and metabolic disorders. Therefore, there is an interaction of factors that might cause dry mouth sensation or hyposalivation. In addition to the chronic diseases already mentioned, some drugs could cause xerostomia as a side effect of the treatment.

In the present study, there was a statistically significant association between medication for the gastrointestinal tract and xerostomia. The elderly individuals who used continuous medication for the gastrointestinal tract were 2.14 times more likely to have the condition of xerostomia (28.6%). A study to assess the side effects of several drugs showed an association between the presence of xerostomia and a digestive tract drug called ondansetron.^6^ However, it has been reported that medications that cause dry mouth sensation could mainly be those with antisialogogic effects, including anticholinergics, antidepressants, diuretics, antihypertensives, antipsychotics and anxiolytics.^22^

In another study, dry mouth sensation was correlated with the drugs used. It was observed that 11 out of 20 medications used by the elderly subjects had side effects of xerostomia and/or hypofunction of the salivary glands, namely: dipyrone, clonazepam, morphine, ondansetron, enalapril, clonidine, metronidazole, tramadol, clindamycin, diazepam and fluoxetine.^6^ Similar results were found in a study by Van Der Putten,^23^ in which the medications that could be the cause of dry mouth sensation or xerostomia were investigated, namely: anticholinergics, antihistamines, antipsychotics, diuretics, sympathomimetics, bronchodilators, benzodiazepines, hypnotics, opioid analgesics, muscle relaxants and antidepressants. According to Perotto et al.,^12^ the symptoms of xerostomia occurred in individuals using antidepressants, anticonvulsants and antihypertensives. According to Villa et al.,^10^ the main cause of hyposalivation and/or xerostomia was the use of medications that included antidepressants, antihypertensives, anticoagulants, antiretrovirals, levothyroxine, supplements and multivitamins, hypoglycemics, steroid inhalers and non-steroidal anti-inflammatory drugs.

It needs to be borne in mind, as a limitation of the present study, that its design was cross-sectional and therefore cause and effect could not be verified, considering that data were analyzed at a single moment. Another limitation that needs to be acknowledged is the fact that memory bias is possible among elderly people when they answer questions. In addition, the variable “chronic diseases and continuous use of medicines” was very broad. Nonetheless, this formed a way of including other diseases and the medicines indicated for their control. Furthermore, this study did not identify risk factors for the self-reported xerostomia analyzed here. If we had been able to evaluate long-term reports on these individuals and their salivary levels, it might have been possible to observe the incidence and factors that could interfere with this condition. Another limitation was the fact that the municipality analyzed is small and we cannot extrapolate the results to other municipalities that are not similar to this one. However, it is important to highlight that the entire population aged 60 years and over that was living in this location participated in this research.

Knowing the causes of xerostomia from self-reports given by the participants in this study will enable implementation of guidance interventions to improve these individuals’ quality of life. However, the data on causality remains uncertain and more information is required in order to be able to reach conclusions regarding the determinants of xerostomia. Nonetheless, the present study is of great relevance and importance for the population surveyed, given that it provides real current data on the elderly people living in this municipality.

## CONCLUSIONS

From the results we obtained in this study, it was possible to conclude that the prevalence of self-reported xerostomia among the elderly people in this municipality is moderate, which corroborates the findings in the literature.

Elderly people with diabetes and other chronic diseases using continuous medications are more likely to have dry mouth. Use of continuous medications for the gastrointestinal tract gives rise to a greater chance of having self-reported xerostomia among elderly people.
